# Estimating Implicit and Explicit Gender Leadership Bias among Primary Healthcare Professionals in Saudi Arabia

**DOI:** 10.3390/ijerph192315871

**Published:** 2022-11-29

**Authors:** Fahad Alzahrani, Khalid Al-Mansour, Ghadah Alarifi, Saad Alyahya, Nasser AlMehaizie, Hanaa Almoaibed

**Affiliations:** 1 Clinical and Hospital Pharmacy Department, College of Pharmacy, Taibah University, Madinah 42353, Saudi Arabia; 2 Department of Studies and Research, King Abdulaziz Center for National Dialogue, Riyadh 13312, Saudi Arabia; 3 College of Business Administration, Princess Nourah bint Abdulrahman University, Riyadh 11564, Saudi Arabia; 4 Riyadh Third Health Cluster, Ministry of Health, Riyadh 11622, Saudi Arabia; 5 King Faisal Center for Research and Islamic Studies, Riyadh 12212, Saudi Arabia

**Keywords:** implicit, gender bias, implicit association test, women leaders

## Abstract

(1) Background: Women have become more influential and powerful; however, implicit bias continues to plague organizations when it comes to women in leadership positions. This study examines the implicit and explicit biases that favor men as leaders among Saudi Arabian primary healthcare professionals. (2) Methods: A secure, web-based survey was administered to primary healthcare professionals. The survey included questions about leadership as well as an Implicit Association Test (IAT) for implicit gender bias. (3) Results: Out of 690 eligible, 448 respondents completed the survey, representing a response rate of 65%. Male residents had a mean IAT score of 0.27 (SD 0.31) and females 0.12 (SD 0.29), both favoring males in leadership roles, and the difference was statistically significant. There was a significant association between gender and gender IAT. In the explicit bias, gender, education, gender of the current manager, and being manager were associated with the gender explicit bias. Explicit bias favoring males in leadership roles was associated with increased implicit bias favoring males in leadership roles. (4) Conclusions: This study found that explicit and implicit gender bias is present among primary healthcare professionals favoring men in leadership positions held by both men and women.

## 1. Introduction

Saudi Arabia’s Vision 2030 emphasizes the importance of inspiring and empowering all members of society to achieve prosperity and progress. To this end, under the current leadership, women’s rights have expanded, and Saudi women are more active than ever before in politics, society, and business [1]. However, Saudi women in leadership positions in many Saudi organizations encounter a different reality when compared to their male counterparts due to various cultural and structural characteristics of Saudi society [2]. The misinterpretation of Islamic instructions and traditions prevailed in Saudi Arabia, limiting the social role of women in the country and similar societies. As a result, their public roles were limited, and they usually stayed at home [3].

Evidence has suggested that within such contexts, women have been less capable of holding leadership positions than their male counterparts [4,5]. According to a study conducted by Al-Halawani, women operate in many parts of government under the umbrella of men, which negatively impacts their performance, and they are limited in their ability to make decisions due to constant intervention by men [6]. Further, the study concludes that women’s ability to lead effectively and make decisions is limited because their authority is lacking due its centralization at headquarters that are controlled by men [6].

The stereotypes about gender are largely based on the different social roles that men and women perform. In particular, women tend to serve as homemakers and caregivers, while men serve as breadwinners [7]. As a result, stereotypes negatively affect societies, especially those undergoing social change towards inclusion and equality, because they reproduce and perpetuate attitudes and behaviors that exclude and oppress people based on their ethnicity, profession, gender, sexual orientation, nationality, accent, language, age, political affiliation, or any other identifier [8,9,10,11].

Leadership traits, such as charisma, decisiveness, and independence are historically associated with men. Women, on the other hand, have historically been associated with traits and attitudes linked to followers [12,13,14]. Consequently, women are still underrepresented in leadership roles in many fields including science, medicine, corporations, and government [15].

There is thus an incongruity between the traits women are expected to possess and the traits that are expected of leaders [16]. This leads to an implicit gender-leadership bias where female leaders are perceived as less likable and more interpersonally hostile than their male colleagues (p. 14) [12]. Consequently, these negative performance evaluations are a considerable barrier to gender equality in the workplace.

Studies suggest that implicit bias may play a role in explaining why men are systematically preferred for positions over women [17]. For example, studies on hiring suggest that men and women show a stronger preference for male candidates (e.g., for working fathers over working mothers)—even when all application materials are identical [18]. One plausible explanation is that those in the position of selecting candidates have unknowingly allowed implicit bias to affect hiring decisions. Similarly, an analysis of 61 studies comparing male and female leaders suggests that bias favors male leaders [19]. Curiously, when women enact a “masculine style” of leadership (e.g., if the female leaders are autocratic and directive), they receive the lowest ratings among men and women leaders, presumably because their agentic behavior conflicts with wider adopted social norms and expectations for women [20].

Bias is defined as a negative evaluation of one group and its members relative to another. Such an evaluation can be expressed directly (e.g., I like men more than women leaders) or more indirectly (e.g., negative nonverbal behavior). Direct or explicit bias also differs from indirect or implicit bias in terms of the underlying process [21,22,23]. Explicit bias requires that the person be aware of their evaluation and be certain that their evaluation is correct in some way. Therefore, explicit bias is measured by asking people to self-report their prejudice [24]. Implicit bias, on the other hand, is an unconscious belief that impacts our behaviors. It develops early in life from the repeated strengthening of social stereotypes and conducts in an unintentional and even unconscious manner [23,25]. It seems to be more prevalent and consistent. It is automatically activated in an unintentional and even unaware manner [23,25,26]. Operating quickly and unknowingly through situational cues (e.g., a person’s skin color, gender, or accent), implicit bias can exert its influence on perception, memory, and behavior. Therefore, implicit bias cannot be measured with self-report survey questions [23,25,27]. Instead, this type of bias can be measured by how quickly people respond to minority-related words or images. Individuals are usually unaware of their own biases [28].

Alsubhi et al. found that women leaders suffer from different challenges, including cultural and organizational challenges, such as gender stereotypes, work-life conflict, and imbalance representation between men and women [29]. According to Al-Asfour et al. (2017) women face some work-related barriers including gender stereotypes and discrimination as well as the lack of professional development opportunities. Also, they found an imbalance between family and work roles and work-pregnancy-related difficulties. Additionally, they argued that work norms mostly reflect the social-cognitive components in Saudi society [30]. The Saudi government vision 2030 considered empowering women and providing leadership opportunities in order to address the previous challenges.

For measuring implicit bias, the Implicit Association Test (IAT), first introduced in 1998, is commonly used and considered to be the most effective tool. The IAT measures how quickly respondents can match social class (e.g., age, gender, ethnicity) to specific attributes (e.g., cooperative, stubborn, and good). Subjects are hypothesized to match a group representative to an attribute more quickly if they connect these factors in their minds, regardless of whether they are aware of this connection. As a result of the fast response times and design of the test, subjects are usually aware that they are making these connections but are unable to manage them [26]. Recent research assessed faculty members’ implicit attitudes toward women and leadership. Based on the results, men were more likely to be associated with leadership than women [31]. Therefore, our primary goal was to investigate implicit and explicit biases favoring men in primary healthcare centers in Saudi Arabia, detect associations between implicit and explicit personal and professional factors, and draw out personal and professional factors that can predict implicit and explicit biases.

## 2. Materials and Methods

### 2.1. Subject Recruitment

In a cross-sectional study, 25 Primary Healthcare Centers (PHCs) workers in five regions of Saudi Arabia were randomly selected to represent the organization of primary healthcare practices in the country. The five regions were Riyadh, Madinah, Aseer, Tabouk, and the Eastern Region. The 15-min, single-session, anonymous survey was hosted on Harvard University’s Project Implicit Web server. Before subject recruitment, all procedures were reviewed by the Saudi Ministry of Health (MOH)’s Central Institutional Review Board (IRB). As a site for IAT research, Harvard University has approved the Project Implicit server. What the study was about was not disclosed to study participants in advance. The study was presented as “An exploration of PHC workers’ knowledge and attitudes regarding gender diversity in leadership” to avoid response bias due to pre-existing knowledge about the topic.

### 2.2. Data Collection

This study was conducted using a multistage random sampling technique on healthcare personnel in Saudi Arabia’s primary healthcare institutions. Geographically, Saudi Arabia is split into the Central, Eastern, Western, Northern, and Southern provinces, and each province has a Directorate of Health Affairs that represents the Ministry of Health. All directorates supervise 2189 primary care centers. We randomly selected five healthcare institutions from each directorate in five provinces for this study. Then, we directed the King Abdulaziz Center for National Dialogue data collectors to every employee at the selected institutions. The data collection began on 27 March 2022 and lasted two months.

PHC workers were invited to participate via a secure website by the study’s primary investigator (F. A.) and three trained research assistants. Additionally, PHC workers gave verbal consent to the survey before it was administered. Participants who consented to participate in the study were given touch-screen devices with links to the study, and they were required to complete them while the research investigators were on duty. As a result, any issues raised during data collection could be resolved consistently and the response rate could be increased. All eligible workers in these primary healthcare settings were recruited. For participants who were unable to attend the first visit, the research assistant was asked to arrange another appointment at their convenience so that the study could proceed at a later time. The total number of invited persons was 690 of whom 448 replied, representing a response rate of 65%. The head of the primary healthcare center in each Health Affairs Directorate coordinated the data collection process in each health directorate. The principal author was available to contact with any questions or difficulties.

### 2.3. Study Measures

To minimize social desirability effects on response, all measures were presented in the following order of appearance: consent page, demographic questions, implicit gender measures (Implicit Association Test), and explicit gender measures (self-reported). For study participants with questions or concerns, the survey included contacting the information of the (P.I) and using KACND devices while collecting data from the respondents to protect their IP addresses and avoid all fears and risks of responding to the study tools associated with that.

*Demographic characteristics*. Primary healthcare professionals were asked to indicate their gender, age, nationality, marital status, years of experience, level of education, education degree source, current and previous manager gender, discipline, and current position.

*Implicit bias.* The IAT, which is a computer-based list of activities that the responder completes, was chosen to measure implicit biases since it has been verified [32]. In this study, the implicit association test was developed by utilizing the implicit association tests established by Harvard University [33]. The IAT is a seven-task format [32].

Table 1 showed that Seven blocks of categorization trials were conducted for each participant. There were four kinds of stimuli in the IAT task, i.e., a test that was administered in this study called “Gender IAT” and that presented Arabic male names (Fahad, Khaled, Mohammad, Saud, Ali) and female names (Leen, Sarah, Noura, Maryam, Ameerah) along with leader’s items (leader, supervisor, manager, director, head), and supporter’s items (subordinate, assistant, supporter, assistant, and aide). All participants completed the IATs in English or Arabic.

The measure of the implicit association test is interpreted as D, ranging from −2 to +2; positive D scores indicate an implicit preference for the group of men as leaders, negative scores indicate an implicit preference for the group of women as leaders, and finally, 0 indicates no implicit preference for either group; higher implicit preference scores are interpreted as an implicit bias against women [34]. Also, the implicit association test is scaled from 1 to 7, with “1” being I strongly prefer men as leaders “4” being neutral, and “7” being I strongly prefer women as leaders. For analyzing Chi-square, we recode the variable to be three levels as “1” prefer women as leaders “2” being neutral (No), and “3” men as leaders.

*Explicit bias.* Explicit scores were obtained a single question asking about feelings toward men and women as leaders. Answer this question “Which statement best describes you” were scaled from 1 to 7, with “1” being I strongly prefer men as leaders “4” being neutral, and “7” being I strongly prefer women as leaders. For analyzing Chi-square, we recode the variable to be three levels as “1” prefer women as leaders “2” being neutral (No), and “3” men as leaders.

### 2.4. Statistical Analysis

Data were analyzed using the Statistical Package of Social Sciences (SPSS), version 28.0. One-sample *t*-tests were used to determine whether the mean IAT score was significantly different from 0, confidence intervals were used to gain insight into the interpretation of significance values, and Cohen’s d was calculated to obtain a standardized effect size to interpret the magnitude of implicit race bias. For Cohen’s d, the effect size is interpreted as: d of 0.2—small effect; d of 0.5—medium effect; and d of 0.8—large effect. The Chi-Square test was used to test the association between both implicit and explicit bias with the personal and professional variables. Ordinal Logistic Regression was conducted in order to detect which variable from the personal and professional variables can separately predict implicit and explicit bias. The P-value for both tests was set at (0.05).

## 3. Results

Table 2 indicated the majority of the participants were between 30 to 39 years old (60%) followed by 40 to 49 years old (20.5%), 18 to 29 years old (13.2%), and 50 to 59 years old (6.3%). Most of the participants were female (58.3%) while males consisted of only 41.7%. The vast majority were Saudis (91.5%) and only 8.5% were non-Saudis. Among the participants, 44% had diplomas, 37.7% had a Bachelor’s degree, 11.6% had a graduate degree, and 6.7% had a high school or less. The study sample consisted of 34.4% nurses followed by 28.3% healthcare specialists, 19.2% physicians, 15.6% administrative, and 2.5% others. Most of the study participants had more than 10 years of experience

According to Table 3, the result of the one-sample *t*-test was significant (*t* = 12.78, *p* < 0.05) which indicated an implicit bias toward men as leaders. Cohen’s d = 0.31.

**Figure 1:**
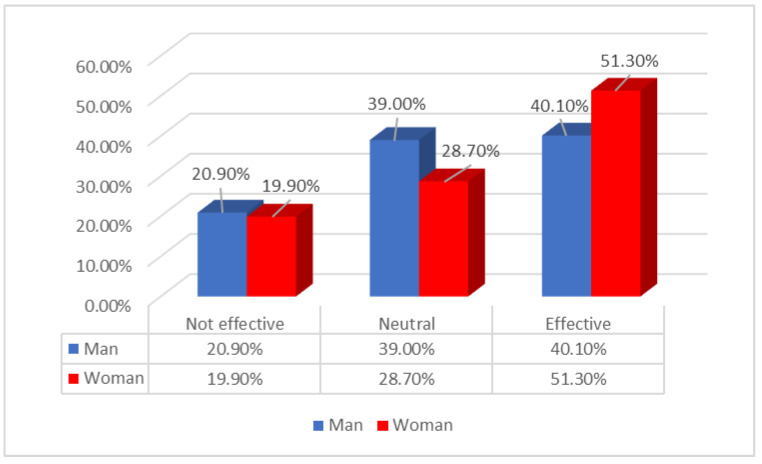
Effectiveness of Women as Leaders.

According to Figure 1, there was only a slight difference between men and women in their opinions about the ineffectiveness of women as leaders (20.9% and 19.9%) respectively. About 10% more men than women tended to be neutral in their opinion of the effectiveness of women as leaders (28.7% and 30%) respectively. It is clear that more women see other women as effective leaders than men.

**Figure 2:**
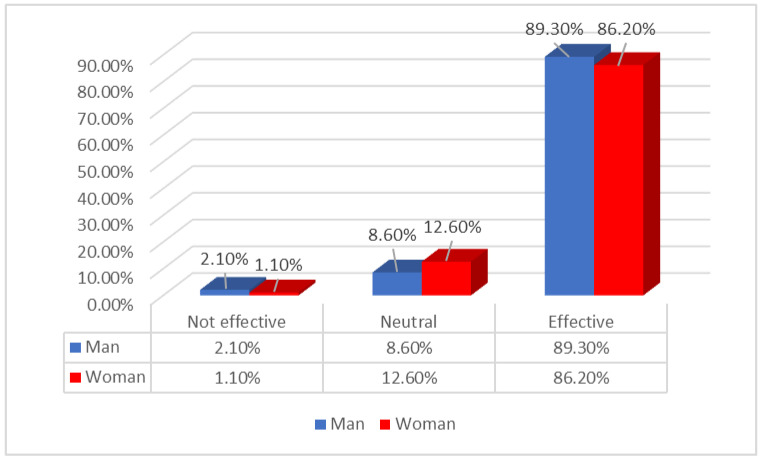
Effectiveness of Men as Leaders.

According to Figure 2, a small proportion of the participants tend to see men as not effective in their leadership: men (2.1%) and women (1.1%). Men tend to be neutral less than women in their opinion of the effectiveness of men as leaders (8.6% and 12.6%) respectively. The vast majority of the participants tend to see men as effective leaders: men (89.3%) and women (86.2%). This result indicated that both men and women were close in their opinion about men as effective leaders.

According to the results in Table 4, only one variable showed a significant result in the Gender IAT. There was a significant association between gender and gender IAT (χ^2^ = 8.6, *p* < 0.05). In the explicit bias, four variables were associated with the gender explicit bias. These variables were gender (χ^2^ = 14.9, *p* < 0.05), education (χ^2^ = 37.2, *p* < 0.05), gender of current manager (χ^2^ = 14.6, *p* < 0.05), and being a manager (χ^2^ = 7.9, *p* < 0.05). It is obvious that men and women tend to prefer men as leaders more than women in both implicit and explicit bias.

Based on the results in Table 5, the model was significant (X = 45.29, *p*= 0.005) and the nagelkerke (R_2_ = 0.101) means the model explains 10.1% of the variance. Among the predictors only two predictors were significant: female (OR = 0.41, *p* < 0.001) and other jobs (OR = 0.28, *p* = 0.39). None of the age groups, nationality, marital status, education, type of living place, years of experience, gender of manager, job, and being a manager showed a significant result to predict implicit bias against women managers.

The results in Table 6 indicate that the model was significant (X = 85.60, *p* < 0.001) and the nagelkerke value R_2_ = 0.184, which means the model explains 18.8% of the variance. Only six predictors were significant: female (OR = 0.57, *p* = 0.008), having a Graduate degree (OR = 0.177, *p* = 0.002), having work experience from 3 to 5 years (OR = 3.54, *p* = 0.006), having more than 10 years of work experience (OR = 3.19, *p* = 0.011), current manager is female (OR = 0.32, *p* < 0.001), and not being manager (OR = 3.18, *p* < 0.001). The predictors of age groups, nationality, marital status, type of living place, and job manager did not reveal a significant result to predict explicit bias against women managers.

## 4. Discussion

The results of the study highlight enduring biases within the healthcare system in Saudi Arabia despite changes in the socio-economic and legal frameworks. Our research has found that despite legal changes and the increasing number of women in the workplace with adequate qualifications, bias against their leadership roles persists. Combining implicit and explicit measures has enabled us to identify key predictors of bias against women in leadership positions within healthcare in Saudi Arabia. While we found some variation between gender, years of experience and specialties, the overall findings show that men and women alike hold both implicit and explicit biases against women in leadership positions in the healthcare workplace.

Gender relationships in Saudi Arabia are influenced by several social and cultural factors. These influence relationships between men and women as well as inter-gender relationships. While Saudi women hold diverse views on gender ideology, traditional gender views naturalize men’s roles as more vocal, and women’s as quiet [35]. This, in combination with the patriarchal culture of the country, creates gender stereotypes of women as predominantly filling roles inside the home rather than in the workplace [36]. The absence of women from driving roles in the last century in Saudi Arabia may be one of the factors that contribute to the tendency of individuals to prefer men as leaders, which led to the formation of stereotypes about the authority to drive pertaining only to men [37], in addition to the fact that Saudi culture until recently was almost devoid of scenes in which women are in a leadership position, as the common pattern for women was to be a housewife [38]. The Saudi vision 2030 has changed the matter completely, as there are women leaders in all sectors. Furthermore, perceptions of appropriate leadership roles and societal gender roles lead to negative perceptions of women in the workplace, who come off as brash and aggressive, or too soft [36].

IAT has been an effective tool in documenting biases within the healthcare system in regard to leadership positions. IAT enabled us to identify gender as a key predictor of bias. The common factor that predicts explicit and implicit bias is gender, indicating a universal bias against women in leadership. This is in part due to sentiments related to the effectiveness of leadership. Our results indicated that while more women than men believe that women are effective leaders, there was significantly more trust in the effectiveness of men in leadership positions. Only 51% of women believed that women were effective as leaders, whereas 86.2% believed that men were effective leaders.

Implicit bias tends to be unconscious bias, where the individual is not only unaware of holding this bias but often holds explicit opinions that contradict these. This is evident in our findings, where women themselves tend to hold a bias against women in healthcare leadership more than men. This could be a result of exposure, where women have predominantly seen men in these roles and attribute their successful careers to the organizational structures they have experienced over the course of their careers within patriarchal workplace culture, a context that can perpetuate women’s status as more closely related to care and men’s to leadership [39,40]. 

Interestingly, in addition to gender in both instances, explicit bias against women in leadership positions had more significant predictors. In the implicit bias, only other jobs and gender were the only significant predictors while the explicit bias had six significant predictors. The significant predictors of explicit bias against women in leadership positions were closely related to education and work experience rather than demographic predictors. Specifically, the explicit predictors were bachelor’s degree, work experience, and experience with managers. These predictors point to experiences of priming within the workplace over time [41], which have led to entrenched opinions related to women’s positions.

Bias against women in the workplace will continue to perpetuate the underrepresentation of women in leadership positions, a phenomenon that is prevalent in most societies [16]. Our results replicate other studies that have investigated gender biases in healthcare in different contexts [39,42]. The prevalence of gender bias in healthcare leadership across cultural settings indicates that while culture may play a role in influencing bias, wider industry biases exist for other reasons, potentially related to the nature of training and employment in the industry, amongst other factors [43].

Despite implicit measures generally resulting in more negative attitudes than explicit measures, our findings indicate that explicit measures indicated more negative attitudes against women in leadership positions. Social psychology research [23,44] and our speculations contradict these findings. To determine whether healthcare professionals, generally, hold implicit gender biases that are slight or strong, and under what conditions implicit gender bias influences promoting institutional change, specifically promotion of women to higher positions, future research will need to study a nationally representative sample of healthcare professionals. According to early research, implicit attitudes and stereotypes may be able to be changed. In order to change implicit attitudes and stereotypes about gender, it is important to stimulate social desirability, suppress known prejudices, and promote counter-stereotypes [22]. According to Dasgupta and Greenwald, individuals were exposed to positive racialized exemplars (e.g., Denzel Washington) and negative white exemplars (e.g., Timothy McVeigh). Using this approach reduced implicit racial bias by 50%, and the effect remained 24 h later [45]. Another study used a standardized 20-min educational intervention to educate faculty about gender implicit biases and strategies for overcoming them. All participants, regardless of age or gender, experienced a positive effect from the intervention regarding implicit biases surrounding women and leadership [31]. For a better understanding of the complex psychologic interactions between healthcare professionals’ implicit and explicit attitudes and stereotypes regarding gender and leadership position, as well as their perceptions of leadership characteristics, future research is needed.

We believe this is the first quantitative study in Saudi Arabia that evaluates health care professionals’ explicit and implicit gender bias. Another strength of this study is that it came after recent efforts to empower women. The result of this study can be generalizable since this study used random sampling. It is important, however, to consider several limitations when examining the results of this study and IAT in general. A second limitation was the limited variety of the study sample, which made it hard to compare implicit and explicit biases among demographic groups. Finally, social desirability might have occurred in this study when some participants may have adjusted their responses in light of the study’s intent.

## 5. Conclusions

Explicit bias appears to be greater than the implicit bias against women in leadership that is prevalent in the healthcare sector in Saudi Arabia and is held by both men and women. Also, it is closely related to experiences of education and work. Work experience thus perpetuates bias against women, perhaps even if these sentiments were not held before working. As more women join the workforce and are appointed to leadership positions, it will be interesting to monitor whether these changes influence biases. Several measures can be implemented to overcome these biases. Future studies should examine levels of transparency of hiring and promotion policies and whether diversity is used as a metric for performance. Furthermore, additional research related to intersectional bias would help uncover additional predictors of explicit and implicit biases towards women’s leadership roles. Qualitative research using different methods of data collection will be beneficial in order to have a deep understanding of the current finding. Follow-up studies will be important in order to detect the changes over time. Policymakers should consider addressing such challenges in order to empower women.

## Figures and Tables

**Table 1:** Gender-Leadership IAT Seven-Task Block Structure.

Block	Function	Stimuli	Items Assigned to Left Key Response	Items Assigned to Right Key Response
B1	Practice	Names of males and females	Female names	Male names
B2	Practice	Words	Leader’s items	Supporter’s items
B3	Test block	Names and words	Female names + leader’s items	Male names + supporter’s items
B4	Test block	Names and words	Female names + leader’s items	Male names + supporter’s items
B5	Practice	Names of males and females	Male names	Female names
B6	Test block	Names and words	Male names + leader’s items	Female names + supporter’s items
B7	Test block	Names and words	Male names + leader’s items	Female names + supporter’s items

**Table 2:** Demographic Characteristics of the Study Participants.

Variable	n	%
**Age (y)**		
18–29 Y	59	13.2
30–39 Y	269	60.0
40–49 Y	92	20.5
50–59 Y	28	6.3
**Gender**		
Male	187	41.7
Female	261	58.3
**Nationality**		
Saudi	410	91.5
Non-Saudi	38	8.5
**Marital Status**		
Single	69	15.4
Married	365	81.5
Divorced	14	3.1
**Education Level**		
High school or less	30	6.7
Diploma	197	44.0
Bachelor	169	37.7
Graduate	52	11.6
**Discipline**		
Physician	86	19.2
Nurse	154	34.4
Administrative	70	15.6
Healthcare Specialist	127	28.3
Other	11	2.5
**Experience (y)**		
0–2 Y	34	7.6
3–5 Y	49	10.9
6–10 Y	109	24.3
More than 10	256	57.1

**Table 3:** One-Sample *T*-Test Result for the Gender Implicit Bias Test.

Variable	t	df	*p*	Mean Differences	Lower 95% CI	Upper 95% CI	Cohen’s d
Implicit	12.78	447	<0.001	0.186	0.158	0.215	0.31

Note. D-scores for participants who completed the gender IAT. Positive scores represent a pro-men (relative to women) bias. Significant results indicate D scores that are significantly different from the no-bias midpoint of 0. *p* < 0.05. Effective size, interpreted as follows: d of 0.2 = small effect; d of 0.5 = medium effect; and d of 0.8 = large effect. *Abbreviation.* N, total respondents for whom we have both Gender D scores.

**Table 4:** Chi-Square for Implicit and Explicit Bias with the Demographic Variables.

Variables	Implicit Preference				Explicit Preference			
Variables	Women	No	Men	χ^2^	Women	No	Men	χ^2^
**Age (Years)**								
10–29	5 (8.5%)	22 (37.3%)	32 (54.2%)	4.51	3 (5.1%)	18 (30.5%)	38 (64.4%)	2.3
30–39	33 (12.3%)	102 (37.9%)	134 (49.8%)		18 (6.7%)	82 (30.5%)	169 (62.8%)	
40–49	11 (12.0%)	26 (28.3%)	55 (59.8%)		5 (5.4%)	27 (29.3%)	60 (65.2%)	
50–59	3 (10.7%)	8 (28.6%)	17 (60.7%)		2 (7.1%)	5 (17.9%)	21 (75%)	
**Gender**								
Male	15 (8%)	53 (28.3%)	119 (63.6)	14.6 *	6 (3.2%)	48 (25.7%)	133 (71.1%)	8.6 *
Female	37 (14.2%)	105 (40.2%)	119 (63.6)		22 (8.4%)	84 (32.2%)	155 (59.4%)	
**Nationality**								
Saudi	45 (11%)	151 (36.8%)	214 (52.2%)	5.8	25 (6.1%)	122 (29.8%)	263 (64.1%)	.33
Non-Saudi	7 (18.4%)	7 (18.4%)	24 (63.2%)		3 (7.9%)	10 (26.3%)	25 (65.8%)	
**Marital Status**								
Single	5 (7.2%)	29 (42%)	35 (50.7%)	5.9	3 (4.3%)	23 (33.3%)	43 (62.3%)	4.7
Married	46 (12.6%)	121 (33.2%)	198 (54.2%)		25 (6.8%)	102 (27.9%)	238 (65.2%)	
Divorced	1 (7.1%)	8 (57.1%)	5 (35.7%)		0 (0%)	7 (50%)	7 (50%)	
**Education Level**								
High School	2 (6.7%)	14 (46.7)	14 (46.7)	7.2	4 (13.3%)	2 (6.7%)	24 (80%)	14.9 *
Diploma	26 (13.2%)	72 (36.5%)	99 (50.3%)		12 (6.1%)	54 (27.4%)	131 (66.5%)	
Bachelor	19 (11.2%)	60 (35.5%)	90 (53.3%)		7 (4.1%)	56 (33.1%)	106 (62.7%)	
graduate	5 (9.6%)	12 (23.1%)	35 (67.3%)		5 (9.6%)	20 (38.5%)	27 (51.9%)	
**Education degree source**								
Saudi Arabia	45 (11.3%)	147 (36.8%)	207 (51.9%)	3.97	26 (6.5%)	120 (30.1%)	253 (63.4%)	1.3
Foreign country	7 (14.3%)	11 (22.4%)	31 (63.3%)		2 (4.1%)	12 (24.5%)	35 (71.4%)	
**Type of living place**								
Village	7 (17.1%)	15 (36.6%)	19 (46.3%)	2.1	1 (2.4%)	14 (34.1%)	26 (63.4%)	5.3
Semi urban city	9 (10.7%)	27 (32.1%)	48 (57.1%)		6 (7.1%)	17 (20.2%)	61 (72.6%)	
Urban city	36 (11.1%)	116 (35.9%)	171 (52.9%)		21 (6.5%)	101 (31.3)	201 (62.2%)	
**Work Experience (Y)**								
0−2	2 (5.9%)	15 (44.1)	17 (50%)	4.3	3 (8.8%)	14 (41.2%)	17 (50%)	7.1
3–5	6 (12.2%)	15 (30.6%)	28 (57.1%)		0 (0%)	16 (32.7%)	33 (67.3%)	
6–10	16 (14.7%)	41 (37.6%)	52 (47.7%)		8 (7.3%)	32 (29.4%)	69 (63.3%)	
More than 10	28 (10.9%)	87 (34%)	141 (55.1%)		17 (6.6%)	70 (27.3%)	169 (66%)	
**Current Manager**								
Male	31 (11.8%)	96 (36.5%)	136 (51.7%)	0.53	5 (1.9%)	62 (23.6%)	196 (74.5%)	37.2 *
Female	21 (11.4%)	62 (33.5%)	102 (55.1%)		23 (12.4%)	70 (37.8%)	92 (49.7%)	
**Previous managers**								
Only man	15 (12.5%)	42 (35%)	63 (52.5%)	0.36	4 (3.3%)	29 (24.2%)	87 (72.5%)	6.9
Only woman	2 (8.3%)	9 (37.5%)	13 (54.2%)		3 (12.5%)	6 (25%)	15 (62.5%)	
Both	35 (11.5%)	107 (35.2%)	162 (53.3%)		21 (6.9%)	97 (31.9%)	186 (61.2%)	
**Discipline**								
Physicians	10 (11.6%)	28 (32.6%)	48 (55.8%)	14.3	4 (4.7%)	29 (33.7%)	53 (61.6%)	3.5
Nurses	19 (12.3%)	66 (42.9%)	69 (44.8%)		11 (7.1%)	45 (29.2%)	98 (63.6%)	
Administrative	11 (15.7%)	21 (30%)	38 (54.3%)		5 (7.1%)	16 (22.9%)	49 (70%)	
Health Specialists	9 (7.1%)	39 (30.7%)	79 (62.2%)		8 (6.3%)	38 (29.9%)	81 (63.8)	
Other	3 (27.3%)	4 (36.4%)	4 (36.4%)		0 (0%)	4 (36.4%)	7 (63.8%)	
**Are you a manager?**								
Yes	5 (12.8%)	17 (43.6%)	17 (43.6%)	1.6	6 (15.4%)	14 (35.9%)	19 (48.7%)	7.9 *
No	47 (11.5%)	141 (34.5%)	221 (54%)		22 (5.4%)	118 (28.9%)	269 (65.8%)	

* = significant result (*p* < 0.05).

**Table 5:** Implicit Predictors.

Variables	Estimate	Wald	OR	*p*	95% CI Upper	95% CI Lower
**Age (y)**						
30–39	−0.066-	0.040	0.936	0.842	0.490	1.789
40–49	0.265	0.418	1.303	0.518	0.584	2.905
50–59	0.450	0.806	1.568	0.369	0.587	4.190
**Gender (Female)**	−0.890-	18.598	0.411	<0.001	0.274	0.615
**Nationality (Non-Saudi)**	0.180	0.136	1.197	0.712	0.461	3.109
**Marital Status**						
Married	−0.272-	0.326	0.762	0.568	0.300	1.935
Divorced	0.126	0.194	1.134	0.660	0.647	1.988
**Education**						
Diploma	0.215	0.225	1.240	0.635	0.510	3.013
Bachelor	0.455	1.054	1.577	0.305	0.661	3.763
graduate	1.045	3.699	2.845	0.054	0.980	8.255
**Ed. Degree source (from a Foreign Country)**	−0.043-	0.009	0.958	0.924	0.396	2.316
**Type of Living Place**						
Village	0.593	2.570	1.809	0.109	0.876	3.732
Semi-urban City	0.353	1.171	1.423	0.279	0.751	2.697
**Work Experience (y)**						
3–5	0.224	0.267	1.251	0.606	0.535	2.926
6–10	0.010	0.001	1.010	0.981	0.442	2.310
More than 10	0.114	0.067	1.120	0.796	0.474	2.646
**Gender of Manager**						
**Current Manager (Female)**	−0.021-	0.013	0.979	0.911	0.674	1.421
Previous Managers (only female)	0.502	1.305	1.652	0.253	0.698	3.906
Previous Managers (Male & Female)	0.031	0.020	1.031	0.887	0.675	1.575
**Discipline**						
Nurses	0.277	0.527	1.320	0.468	0.624	2.790
Administrative	0.132	0.097	1.142	0.755	0.496	2.626
Health Specialists	0.439	1.706	1.552	0.191	0.803	3.000
Other	−1.274-	4.250	0.280	0.039	0.083	0.939
**Current Role (Not Manager)**	0.186	0.335	1.205	0.563	0.641	2.265

The model (X = 45.29, *p* = 0.005) and nagelkerke (R_2_ = 0.101).

**Table 6:** Explicit Predictors.

Variables	Estimate	Wald	OR	*p*	95% CI Upper	95% CI Lower
**Age (y)**						
30–39	−0.535	2.446	0.586	0.118	0.300	1.145
40–49	−0.571	1.779	0.565	0.182	0.244	1.307
50–59	−0.441	0.679	0.643	0.410	0.225	1.837
**Gender (Female)**	−0.559	6.958	0.572	0.008	0.377	0.866
**Nationality (Non-Saudi)**	−0.342	0.518	0.710	0.472	0.280	1.802
**Marital Status**						
Married	−0.074-	0.021	0.929	0.885	0.344	2.508
Divorced	−0.246-	0.703	0.782	0.402	0.440	1.389
Education						
Diploma	−0.547	1.274	0.579	0.259	0.224	1.496
Bachelor	−0.889-	3.456	0.411	0.063	0.161	1.050
graduate	−1.730	9.478	0.177	0.002	0.059	0.533
**Ed. Degree source (from a Foreign Country)**	0.689	2.431	1.992	0.119	0.838	4.739
**Type of Living Place**						
Village	0.477	1.703	1.611	0.192	0.787	3.297
Semi-urban City	0.181	0.326	1.199	0.568	0.644	2.233
**Work Experience (y)**						
3–5	1.264	7.705	3.541	0.006	1.450	8.648
6–10	0.741	2.990	2.098	0.084	0.906	4.859
More than 10	1.159	6.388	3.185	0.011	1.297	7.822
**Gender of manager**						
**Current Manager (Female)**	−1.141	32.191	0.320	<0.001	0.216	0.474
Previous Managers (only Female)	0.538	1.247	1.713	0.264	0.666	4.405
Previous Managers (Male & Female)	−0.169-	0.573	0.844	0.449	0.544	1.309
**Discipline**						
Nurses	−0.001	0.000	0.999	0.998	0.479	2.085
Administrative	−0.210	0.264	0.810	0.607	0.363	1.808
Health Specialists	−0.270	0.685	0.763	0.408	0.402	1.448
Other	0.263	0.152	1.301	0.697	0.346	4.885
**Current Role (Not Manager)**	1.157	12.191	3.182	<0.001	1.661	6.093

The model (X = 85.60, *p* < 0.001) and nagelkerke (R_2_ = 0.184).

## Data Availability

Data are available upon reasonable request by contacting the corresponding author.
